# St. George's Hospital

**Published:** 1829-10-01

**Authors:** 


### ST. GEORGE'S HOSPITAL.

### I. SLOUGHY ABSCESS OF THE CELLULAR MEMBRANE OF THE THIGH AND ULCERATION OF THE CARTILAGES OF THE KNEE-JOINT.

The cellular texture of the lower part of the thigh is peculiarly liable to unhealthy inflammation and suppuration. We are not aware that this affection is described in a particular manner by authors, but it well deserves to be so, on account of its frequent occurrence, and generally fatal termination. The subjects of the disease are mostly of a weak and scrofulous habit, and persons exposed by their occupations to atmospheric changes would appear to suffer more than any others. The knee-joint has been implicated in all the cases which we have observed, but whether in a primary or secondary manner it is difficult precisely to determine. The characters and progress of the affection will be better illustrated by one or two cases, than by any description we can give.

*Case* 1. Eliza Goddard, ætatis 33, was admitted on the 18th of March, under the care of Mr. Hawkins, with much diffuse swelling about the knee, the upper part of the leg, and the lower portion of the thigh. The skin was not discoloured in the least degree; the leg was œdematous; the thigh was not, but rather resembled in appearance the phlegmatia alba dolens, being somewhat elastic under pressure. Above the patella on the inside of the thigh was the opening of an abscess, which discharged a large quantity of pus. The tenderness of the parts on pressure was excessive, the pulse very quick and weak, the constitution greatly disturbed.

The swelling had first appeared about five weeks previously, and the abscess had been opened in the course of a week from the commencement of her complaint. She was ordered by Mr. Hawkins a pint of porter daily and an effervescing draught containing the carbonate of ammonia, spirits of nitric æther, and laudanum, every six hours. She also took an opiate at night.

On the next day, another opening was made below the knee and half a pint of bloody pus discharged with much relief. The bone was not exposed, nor did the probe pass fairly into the joint, but it went for some distance up the thigh betwixt the muscles. Bark and red wine were ordered, and on the 24th one or two incisions were made in the leg and sloughy cellular membrane exposed. The pulse was 140—the tongue generally clean, but dry.

30th. Her health is on the whole improved; she has less irritation and is not become materially weaker. The abscess below the knee discharges rather less, but that on the inside of the thigh passes up for some distance beneath the extensor muscles. The opening in question was enlarged that the escape of matter might be free, and a counter opening was also made through the vastus externus. On the 31st, she complained of great sickness and pain at the præcordia, the discharge was copious and of bad quality, the depression great. Brandy was given, and next day a draught containing the liquor opii sedativus. Diarrhœa succeeded, and was checked by chalk mixture, but the patient was fast wearing out with the constant discharge, the hectic, and the excruciating pain in the limb. More than one consultation was held amongst the surgeons, but the general disturbance of the constitution was deemed a perfect bar to amputation, which was therefore not proposed. A considerable portion of the shaft of the femur could be felt by the probe, exposed and rough; aphthæ formed about the mouth, and on the 14th of April the patient sank, in spite of vigorous support.

*Sectio Cadaveris.* The body was extremely emaciated. The several incisions about the knee exposed the cellular texture in a dark and sloughy state, and the one on the inside of the thigh passed through the vastus internus down to the inner condyle, and into the knee-joint. The femur, up to the middle of its shaft, or even higher, was completely isolated from the soft parts around, which formed the walls of one large sloughy abscess. The capsule of the knee-joint and lateral ligaments were almost entirely destroyed — the joint itself communicated with the cavity just described by means of the destruction of its soft parts, and with the external air through the medium of the incision on the inside, and another on the outside of the thigh. The cartilages of the knee-joint were totally destroyed by ulceration, and the bone discoloured, but not carious. The patella appeared to be softened; the joint of the head of the fibula was affected in a similar manner with the knee, but not to so great an extent. The femoral vessels though actually forming part of the walls of the sloughy abscess in the thigh were pervious and healthy. The viscera were not examined.

*Case* 2. John Foster, æt. 17, was placed under the care of Mr. Hawkins, on the 25th of March.

He had pain and tenderness in the left ham, and in various parts around the knee, accompanied with a little fulness of the parts. The limb started much, and the pain was increased upon pressure, but what was singular, he could walk about at times as well almost as if no uneasiness whatever existed. There were febrile symptoms, as brown tongue, parched lips, and so forth. In the course of a few days the fulness about the knee had increased, and the synovial membrane seemed to be distended. There was not much pain or tenderness experienced, except when pressure was made in the course of the popliteal nerve. Leeches and a poultice were applied, but the fulness of the lower part of the thigh was soon converted into evident swelling with a sense of deep-seated fluctuation, and much constitutional disturbance.

*Salines with antimony, and an opiate at bed time.*

The fluctuation became more distinct, and on the 17th of April a seton was passed from the inside of the thigh through the ham, taking care to avoid the popliteal nerve, which lay to the outside of the seton thread. Some purulent matter of very offensive quality followed the introduction of the seton. Profuse discharge and irritative fever of a nature to preclude, in the opinion of Mr. Hawkins, any operation, were now set up; a large counter-opening was required in the under part of the thigh, which exposed the femur rough and dead; the patient progressively sank beneath the irritation, and on the 25th or 26th inst. he expired.

*Sectio Cadaveris.* We had not the opportunity of witnessing the examination of the body, but we understand from those who did, that the appearances in the limb were similar to those in the former case. The cartilages of the knee-joint were in a state of ulceration, and a *foyer* of pus extended upwards along the femur, which was bare, and in some parts dead. The vessels in the ham, and especially the nerve, were in contact with the matter, but the cavity of the artery and vein was free, and the coats of the vessels were not observed to be inflamed.

The case altogether bears a striking similarity to the first, and also to another which occurred in the practice of Mr. Keate.

*Case* 3. George Ranscombe, æt. 34, admitted on the same day as the preceding patient, under the care of Mr. K. The lower half of the right thigh was occupied by an extensive and deep-seated abscess, which had commenced with swelling four weeks previously. He stated that at different times, but not lately, portions of bone had exfoliated from the femur. He was much worn down, of scrofulous aspect, with dry tongue, small and weak pulse, and hectic flush upon the cheek. Openings were made at each side of the thigh at its inferior part, and a considerable quantity of purulent matter discharged. A poultice was applied, and anodynes with bark and other medicines of that description freely given. On the 28th, a counter-opening was made on the outside of the thigh, and the bark in its ordinary form disagreeing, the sulphate of quinine was prescribed in its stead and with good effect. The improvement, however, was of very short duration, for in a day or two the patient fell back into his former state of hectic fever and excessive irritation, under which he sank on the 21st of April.

*Sectio Cadaveris.* The body was attenuated to a great degree. Nothing unusual was observed in the chest or the abdomen. The femur from about three inches below the trochanters to the knee-joint, was separated from the soft parts entirely and placed in the centre of one large sloughy abscess completely encircling the shaft of the bone for the space described. The bone itself was more or less deprived of its periosteum, and towards the condyles had several carious or ulcerated spots. In the neighbourhood of these new ossific matter was deposited, and in one part in particular, a good example of incipient necrosis was presented. The semi-lunar cartilages of the knee-joint were ulcerated, but the rest of its structures were comparatively unaffected.

We have thought it right to detail these cases because they may serve to describe an affection worthy consideration, both on account of its frequency and danger. The cellular tissue around the femur at its lower part would seem to be particularly prone both to chronic and acute inflammation. When the former takes place, necrosis is the consequence; when the latter, the patient is too often worn out by the hectic fever and great irritation set up. In all the cases that we have seen, the knee-joint has been more or less affected, though sometimes indeed at an earlier period than at others. When the inflammation is rheumatic, calomel and opium appear to hold it more in abeyance than any other means with which we are acquainted, but even these remedies frequently fail. In the common forms, such as the cases above detailed, we are not aware that any specific measure has proved of much avail, and very much fear that it will not. The question of amputation will no doubt present itself to the mind of the surgeon, and a number of circumstances peculiar to the individual case must guide his decision upon this point.

### II. NOTES OF THE DISSECTION OF A PATIENT WHO DIED OF APOPLEXY—STATE OF THE HEART AND ARTERIES.

We are induced to publish the notes of this dissection, because they support important views both in pathology and practice. The patient was a woman scarcely 40 years of age, who died under the care of Dr. Seymour, and was examined on the 18th of May last, in the dead-house of the hospital. The body was remarkable for its fulness of contour and enbonpoint.

*Head.* Nothing particular was noticed about the calvarium, dura mater, membranes on the surface of the brain, or that surface itself. On carefully slicing down the right hemisphere to the lateral ventricle, the latter was found filled with bloody serum, and contained in its cavity a clot of recent blood. The coagulum was mainly in the body of the ventricle, and passed for some depth into its descending horn, but not into the anterior or posterior. Its seat was partly in the substance of the thalamus opticus, partly in the corpus striatum, but more in the former than the latter. The clot was about the dimensions of a walnut, and had made its way into the cavity of the ventricle by breaking up the connexion between the thalamus and striated body. The left ventricle was distended by much bloody serum, but no coagulum was present. The arteries at the basis of the brain, especially the basilar and its chief ramifications, had osseo-cartilaginous depositions in their coats. No rupture of any one large vessel was detected.

*Heart.* The parietes of the left ventricle were full an inch in thickness, but its cavity was not much enlarged. The muscle of the right ventricle was not above a line in thickness, and tore with the slightest force. The heart on the whole was larger than natural. The mitral valve was thickened, and gritty depositions were felt in its substance. Ossification was commencing at the attached border of the semilunar valves of the aorta, and the coronary arteries were beginning to be ossified. The aorta itself, or at least its arch, for the rest was not examined, was free from disease.

Nothing unusual was seen in the abdomen, save that a small fleshy tubercle grew from the external surface of the uterus.

As the effusion of blood was found in the right ventricle, it may be well to state that the patient during her attack had laboured under hemiplegia of the opposite side, as generally happens. Our object, however, in noticing the case was not so much in reference to apoplexy, as the connexion betwixt the condition of the heart, and that of the arterial tubes. The latter were ossified, or beginning to be so, and the left ventricle of the former was hypertrophied in its parietes, even somewhat dilated in its cavity. This is not a matter of chance, nor a mere coincidence; on the contrary, we believe that the two will commonly be found to co-exist. Which is the cause and which the effect is, of course, very difficult of absolute proof, although we believe that the state of the heart is the secondary affection, and only stands heir to that of the arteries. These tubes becoming rigid, the circulation is virtually impeded, and thus an additional onus is thrown on the left ventricle, which it meets, as muscles do elsewhere, by an increase in the volume of its fibres and walls. In the interesting case related by Dr. Johnson in the last Number of the Journal, this condition of the circulating organs had occurred, and whatever explanation our readers may adopt, the frequency of such occurrence is beyond a doubt. In the majority of cases the ventricle is simply hypertrophied without dilatation of its cavity, and the right chambers of the heart are frequently but little altered, although their condition is certainly subject to variation. Thus, in Dr. Johnson's patient, the cavity of the right ventricle was extremely small, and in another which we lately saw it was unusually large, but in many we believe that the fact is as we stated, and the alteration comparatively little. As we mean in a future report to return to the interesting subject of diseases of the heart, we shall say no more upon the point at present.

III. Ulcerated Nævus at the Bend of the Arm.

On the 23d of April, a child was brought for the inspection of Mr. Keate with an irregularly shaped ulcerated nævus at the bend of the left elbow-joint in front of the arm. The nævus had existed since birth, had not subsequently undergone much increase of volume, was about the dimensions of a half-crown piece, but much more irregular, and a considerable part of its centre had been eaten away by the ulcerative process. The skin had apparently merely given way in the first instance, in consequence of the motions necessarily taking place in the joint, and not from ulceration, though the latter was speedily grafted on this accidental fissure. A straight pasteboard splint, and afterwards a wooden one, were applied to the back of the arm, and the two bound together by some turns of a roller above and below the ulcer. The limb being thus kept quiet and immoveably extended, various dressings and a poultice were employed with the view of healing the sore. The attempt, however, not proving successful, the potassa fusa was applied to the surface of the parts, so as to induce a moderate slough, the close proximity of the brachial vessels forbidding the formation of a deep one.

The slough came away in two or three days, but a good deal of swelling at the bend of the arm and some general disturbance of the system ensued. These gradually passed away, and the sore looked clean, although a sinuous opening led down to a cavity containing matter beneath the granulations. Compression was applied, the sore diminished, and the last time we saw the little patient, it was nearly healed.

### XXXVIII.

### ST. GEORGE'S HOSPITAL.

#### DISEASES OF THE HEART.

The present season has been and is peculiarly rife in diseases of the heart, diseases that present very marked and distinctive characters, but yet would appear to be ill understood by the mass of the profession. In less than three months, upwards of 18 cases of organic alteration of the heart have been *admitted* within the walls of St. George's Hospital, half of which have proved fatal during that period, and afforded opportunities on an extended scale, of comparing symptoms with post-mortem appearances. It is a circumstance much to be deplored, that the great majority of these patients had been treated, before their application at the hospital, for affections widely different from that which was the real malady, the fons et origo of their ills. Many had been bled and depleted for inflammation of the lungs. Still more had been made to swallow a farrago of remedies directed against *asthma*, the universal scape-goat for these diseases. The errors to which we are now alluding, were not *all* committed by practitioners of no reputation, but more than one mistake in the catalogue may be laid to the door of individuals of no inconsiderable eminence. However, this is an unpleasant topic, and we gladly pass on to our more immediate province, the details of some of the cases themselves. The reader will find allusions not unfrequently made to the physical signs of the disease, and we trust we may be permitted to add without offence, that the degree of accuracy they confer on the diagnosis of most cases, and of positive certainty on many, is extremely gratifying.

CASE 1.—*Hydrothorax on both sides—Tubercles in Right Lung—Hydrops Pericardii — General Dilatation of Heart, with Hypertrophy of Left Ventricle—Pus in Polypi.*

J. Snowden, æt. 34, a painter, admitted May 20th, under the care of Dr. Seymour.

Face puffy, sallow, and leuco-phlegmatic—cellular membrane of scrotum and legs œdematous—some little fluid in the belly. Breathing hurried and short, but not painful — dyspnœa on exertion or ascent — orthopnœa — difficulty of lying on left side—broken sleep and frightful dreams—sense of beating and oppression at the heart, the action of which is felt by the hand to be rapid and tumultuous, but apparently not close beneath the ribs, as if there was fluid interposed. Pulse 140, not hard nor bounding, but a little full and sharp—tongue moist, with whitish yellow fur on its surface—appetite indifferent—urine scanty—bowels open.

Was suddenly attacked two months ago, whilst walking against the wind with shortness of breath and fluttering at the heart. Before this, however, he noticed that his face was becoming swollen. About six weeks ago his feet swelled, when his breathing became easier — body has swollen within the last few days. Has not been able to follow his employment since the commencement of the present illness, but has not been uniformly bad all the time. Has had several attacks of the colic of his trade, and been almost always ailing—never laboured under rheumatism. Has been accustomed to drink spirits.

*V. S. ad* ℥x. *vesp. Cras mane, Elaterii* gr. i. *Hyd. Submur.* gr. ij. ℳ. *ft. pil.*

Next day a more particular examination was made, and the liver, especially its left lobe, felt to be enlarged. Dr. Seymour concluded from the symptoms above detailed, that the heart was dilated, whether with or without hypertrophy of its walls, he could not at that time make up his mind; and that there was hydrops pericardii, with fluid in the pleuræ, and old disease in the lungs. On applying the stethoscope in the region of the heart, the action of its right chambers was heard rather loud and clear, without impulsion, over a considerable space. The left ventricle, on the contrary, had not only extent of pulsation, but more impulsion than natural. It was curious that the ventricular systole was not directly communicated to the instrument, but produced a kind of oscillatory movement, as if there were fluid interposed between them. The inferences drawn from the above indications were—general dilatation of heart—dilatation with hypertrophy of left ventricle—hydrops pericardii. There were also reasons to believe from the auscultic phenomena that the lower lobes of both the lungs, especially the left, were irrespirable, either from water in the pleuræ or consolidation of their texture, probably from both causes. The pulse at the wrist was rather jarring but not full, indeed it was the reverse.

℞ *Potass. Acet.* ʒj.—*Vin. Col.* ʒss. *Aq. Menth. Vir.* ʒx. *Tr. Scill.* ♏. x. ♏. *ft. haust. bis quotid. Potus supertartrat. potass. ad* ℈j. *in die. Rep. Pil. Elater. alternis auroris.*

We shall not take up our reader's time by the daily nor even the weekly details of this case, but shall merely allude to the more important events that marked its progress. On the 31st (little alteration on the whole had taken place) he was ordered *pil. hyd.* gr. iij. *pil. scill.* gr. j. *pulv. digital.* gr. j. *ter die*, and on the 1st of June a *blister* was applied to the chest, in consequence of the cough which was gradually growing on him, accompanied with expectoration of much frothy mucus. On the 3d, the effusion into the limbs being greater, and the cough severe, he was *bled* to ten oz. and part of the blood shewed the buffy coat. The pulse was weak, and oppressed. In the evening he took the *bolus hydrargyri c. scill.* On the morning of the 4th was *bled* again to twelve oz. and took the *bolus*, a second time, that evening.* On the 6th, he appeared in a critical state—the depression was extreme, and a lurid blush with tumor looking like something intermediate between erysipelas and carbuncle, occupied the nose and cheek on either side of it. The former medicines were discontinued, and *quinine draughts* with *opiates* substituted for them. The patient gradually rallied; the erysipelas, if such it was, passed away by degrees; and on the 11th, the symptoms were much the same as they had been before their recent exacerbation.

The sputa, however, now began to assume a lumpy and suspicious purulent appearance, the skin was always cold, the pulse very shabby. He was noticed to be constantly dozing in the sitting posture, his short sleep being broken every now and then by a convulsive start. The treatment consisted in *cabbage leaves* to the legs, and an *opiate* with the *oxymel of squills* and *spirits of nitric æther* twice daily; for the quinine, appearing now to disagree, was left off after the 21st. He was *bled* to ten oz. on this day. Emaciation was making progress—the effusion into the external, and probably also into the internal parts disappearing, and the strength declining—but what was singular, the cough subsided, and the expectoration about this time almost disappeared. On the 9th July we find him taking port wine and astringents, diarrhœa with aphthæ in the mouth having supervened, but, in spite of all the support that could be given, he sank on the 16th. On applying the stethoscope to the præcordia, during the last week of his illness, the impulsion of the heart was communicated more directly to the instrument, and the peculiar oscillatory movement, noticed on the former examination, was gone. This circumstance seemed to shew that the hydrops pericardii had materially diminished. The lungs after the first week had not been examined by auscultation, as the patient had evidently no chance of recovery, and it was not wished to disturb him unnecessarily.

SECTIO CADAVERIS. Body emaciated to an extreme degree.

*Thorax.* Two pints or more of yellow serum, with shreds of lymph in left pleural cavity, and a few slight adhesions anteriorly above—rather more serous effusion in right cavity, with strong adhesions in front and above, so arranged as to confine the fluid in its inferior portion. Pleuræ on either side exhibiting arborescent injection of their vessels, and patched with old lymph in various places.

Left lung œdematous throughout upper lobe, but crepitating well—lower lobe more *engoué*, and its inferior half so consolidated as to sink like lead in water—thin frothy fluid in bronchi, the mucous membrane which reddened. Great part of right lung, especially the upper lobe, infiltrated with tubercles, collected together in circumscribed patches, with condensation of the neighbouring parenchyma. One of these tubercular masses particularly noticed between the upper and middle lobes, and displaying in its centre a vomica large enough to contain a small egg—some other smaller vomicæ at the apex of this lung. Lower portion of the lung

* This bolus is a hospital formula; it consists of twenty-four grains of blue pill and six of recent squills.

resembling that of the left, and sinking, like it, heavily in water—about the middle of the lung a little diffuse suppuration.

Pericardium containing a couple of ounces of bloody serum—pericardium itself injected with blood-vessels, but not thickened or spread with lymph.

Heart very large—auricles and right ventricle passively dilated in their cavities—left ventricle both dilated in its cavity and hypertrophied in its wall—valves and great vessels healthy. Heart filled with loose coagula—near the apex of left ventricle, a small mass of what looked like lymph, surrounded and blended with the black coagula, and *containing in its centre a creamy fluid, having all the general properties of pus.* In the interstices, between columnæ carneæ, numerous small compact masses of lymph, most of them, when broken, found to contain in their centre the same sort of purulent fluid as the larger one.

*Abdomen.* Liver enlarged, and as hard almost as cartilage—its appearance, upon section, something between that of nutmeg and granite—pancreas excessively hard—kidneys remarkably diminutive, and the left containing one or two cysts, filled with dirty serum.

*Head* not examined.

Whether the tubercles in the right lung were formed, in whole or in part, between the 20th of May, when the patient was admitted, and the 15th of July, when he died, is a question which we shall not pretend to moot. That the vomicæ appeared in that interval, we think there can be no doubt, from the general symptoms and the stethoscopic indications in the first instance. It will scarcely fail to be perceived, that the tubercles were not of the common scrofulous character—that they were agglomerated into masses—in fact, that they presented a specimen of the "tuberculous infiltration." The largest vomicæ, too, were not at the apex of the lung, as in phthisis pulmonalis, but in the centre, where the mass of infiltration was greatest between the upper and the middle lobes. Is it possible that this is the species of tubercle developed by inflammation? We know of some curious facts on this subject, but it is yet involved in mystery and difficulty. We mentioned that there was pus in the lymph or fibrine deposited in the ventricle. We do not ourselves imagine that it was the result of inflammation; on the contrary, we conceive it to have been produced by some decomposition of the polypus itself.* The patient, it should be recollected, was very long in dying. Dr. Seymour possesses a most beautiful drawing, by Mr. Perry, the artist, of the apex of the ventricle, with these abscesses (to use an improper term) in situ.

Dr. Seymour remarked, in his clinical lecture, that, on the admission of this patient, it was evident he laboured under an incurable state of disorganization of the heart, together with chronic disease of the lungs, and the symptoms were so severe as to threaten almost immediate death. The dropsy of the cellular membrane was the result of these. All that could be done for such a state of things was to alleviate the symptoms of distress, and endeavour to procure the absorption of the fluid which was producing so much oppression. For this purpose, small bleedings were had recourse to, not only to relieve the congestion in the lungs, but likewise to promote the absorption of the fluid. The elaterium was used principally to empty the cellular membrane, through the medium of increased evacuation of fluid from the bowels, for which purpose it is the most powerful of all the remedies we possess. The effect was produced in some degree, the fluid being diminished, and corresponding comfort, as more quiet sleep, more facility of lying in the horizontal posture, produced. A low attack of

---

* On looking at Laennec's work, we find that he has given a very accurate description of these bodies, under the term of *globular excrescences.* All the patients so affected, whom he had seen, have remained "in a dying state for several days or even weeks." He does not imagine that they are formed by inflammation. The whole passage is very interesting, and we refer our readers to pp. 668 *et seq.* of Forbes' translation, third edition.

inflammation having occurred, small bleedings were again had recourse to, with mercury and squills, and the relief appeared to be great, when he was attacked with erysipelas and great depression of strength. The medicines were obliged to be changed, but, on the subsiding of the erysipelas, the former plan was repeated, until the symptoms of weakness rendered it improper. The first bleeding was employed with great caution, and only repeated from the great relief experienced and expressed by the patient, whose respiration became, for a time, greatly relieved. To those who witnessed this case, it appeared manifestly to afford decided temporary comfort.

CASE 2. *Peripneumony on Left Side—Encysted Effusion into Left Pleural Cavity—Passive Dilatation of the Heart.*

Patrick Gillon, æt. 43, a hawker, admitted, June 24, under the care of Dr. Chambers. He gave the following account of his complaints both present and antecedent, an account calculated to deceive the enquirer.

Pain in the left side of the chest, increased on deep inspiration—imperfect expansibility of that side—difficulty of lying in any position, except on the affected side—a little cough, with expectoration untinged with blood—occasional pains "all over him"—no œdema of the legs—pulse very shabby—urine high-coloured—bowels confined.

Spat blood last Winter, to the amount of two quarts at one time, but whether discharged by vomiting or coughing he cannot distinctly say. Present illness began only a fortnight ago, with general pains and cramps, together with the pain in the left side of the chest and the cough. Knows no cause for his complaint.

There was little here to indicate heart disease, much to establish peripneumony on the left side.

*V.S. ad* ℥xvj. *Haust. salin. cum Potass. sulph.* ʒj. *4tis horis. Diæta parcissima.*

The blood drawn did not seem to have flowed well; it was not cupped. The pain in the chest was relieved but not removed. The bleeding was repeated to ten ounces with more relief, and on the 26th, there was no pain on taking a full inspiration, although there was some in the left side without it—cough rather frequent and short—expectoration of frothy consistence mixed with greenish, clear, muco-purulent matter—pulse soft, irregular, and inclining to intermit.

The lower half of the left lung afforded only bronchial respiration, and the sound over the same extent was extremely dull on percussion, shewing that the lung was consolidated, and probably hepatized for that extent.

*Emp. canthar. lat. sinist.* ℞. *Cal.* gr. ij. *Op.* gr. ss. *ter die. H. sal. sine pot. sulph. (It purged him.)*

29th. Has pains all over him, especially in head and left shoulder—pain on deep inspiration or coughing in lower part of left side and epigastrium—cough troublesome, but not more so at night than by day—expectoration not great in quantity, clear, and chiefly frothy—pulse rapid and frequently intermitting. By *auscultation*: respiration heard louder than natural all over right side, and especially under right clavicle; respiration bronchial throughout *upper* parts of *left* lung, but is gradually obscured and finally lost in the lower lobe. This showed that *the lower lobe was almost totally impervious to air, and the upper only receiving it in the larger bronchial ramifications.* The patient progressively improved till the 6th, when in consequence of the mouth being slightly sore, he only took the *calomel* and *opium* at night, with a dose of *castor oil* every other morning.

On the 10th July he complained of violent and frequent action of the heart in epigastrio, and of the vessels in the neck—pulse very small and quick—urine scanty and high-coloured—bowels confined. Dr. Chambers now suspected organic disease, most probably dilatation of the heart, and ordered a *belladonna plaster* to the præcordia, *a scruple of the compound senna powder* every morning, and *camphor mixture* with *half a drachm of tincture of hyosciamus* thrice daily. The former medicines were discontinued. Some few days after this, on examining the heart with the cylinder, the contractions of the auricles and ventricles were heard over a considerable space, with slight and but a slight impulsion in the left ventricle. The sound was clear, the actions of the chambers were at times irregular. These signs appeared to indicate *passive dilatation of the cavities of the heart—dilatation of the left ventricle, with perhaps slight hypertrophy of its parietes.*

We need scarcely pursue the details of the case; suffice it that we find dyspnœa, œdema of the legs, and puffy face, shortly make their appearance—that the cough and expectoration diminished —but the pulse grew weaker and more decidedly intermitting. On the 13th he was ordered the *sp. æth. nit.* and on the 15th, being griped by the senna, *rhubarb* and *aromatic confection* were substituted for it. On the 16th, the latter was omitted also, and an *opiate* with *cardamoms* prescribed.

On the 17th, he suddenly became worse, and indeed appeared to be dying. He required to be propt up in the sitting posture, inclining to the left side—the face and extremities were cold and blue—the breathing rapid and oppressed—the pulse at the wrist almost imperceptible—the conviction that of approaching dissolution. No respiration was detected by the stethoscope in the lower part of the left side of chest, where fluid appeared to be effused. Warm *wine and water, aromatic and opiate confection in camphor mixture, and compound æther mixture,* were successful in reviving him from this state of alarming prostration, and he continued from the 18th to the 22d, almost in as comfortable and quiet a condition as before the late attack. On the 22d, the bowels being rather confined, he was ordered to omit the opiate confection, and castor oil was prescribed for the next morning. When that arrived, however, he suddenly became worse, and died in the course of a very few minutes.

SECTIO CADAVERIS. Body much emaciated.

*Thorax.* No fluid, and only some old lax, cellular adhesions between pleuræ on right side. Pleura costalis on left side much thickened and closely adherent over upper lobe of lung to pleura pulmonalis, except in one part, where a small circumscribed cavity was left between them, containing a little fluid. Over lower lobe, a much larger circumscribed cavity between pleuræ, in which was a pint and a half of bloody fluid. Pleura pulmonalis here coated with layers of lymph, chiefly ancient.

Whole lower lobe of left lung consolidated by previous inflammation; lower lobe imperfectly crepitous, engoué with blood, easily lacerable, and presenting a few scattered, crude tubercles. Right lung generally gorged with blood, but crepitous, and swimming in water—some scattered crude tubercles in upper lobe.

Pericardium containing little fluid. Heart much dilated in its cavity, and walls of right chambers flabby in the extreme—left ventricle solid to the grasp, its cavity enlarged, its parietes increased in thickness rather than diminished, but not of bright, fleshy, muscular colour. Valves sound—lining membrane of heart and great vessels tinged of dark colour by the blood, which was in quantity, and loosely coagulated.

*Abdomen* contained a little clear serum, but its contents were universally sound.

*Head* not examined.

The family likeness between the two preceding cases is not obscure. Both were examples of passive dilatation of the heart, with slight increase of substance in the walls of the left ventricle —both shewed a disposition to tubercular deposits in the lungs—both afforded specimens, the latter far the best, of the circumscribed pleural effusion, of Bayle and Laennec. The differences between the cases will be readily appreciated by the reader, especially the greater quantity of dropsy in one case than the other, and the fact, perhaps explanatory of the circumstance, of liver and kidney affection in the former, but not in the latter. Our limits oblige us to stop, but in a future report we shall detail some cases of active enlargement of the heart, or hypertrophia with dilatation.

### CLINICAL REVIEW.

### LI.

### ST. GEORGE'S HOSPITAL.

### Diseases of the Heart.

Every day convinces us more strongly of the prevalence of diseases of the heart and great vessels, of the general ignorance that prevails in the bulk of the profession respecting them, and of the lamentable consequences resulting from that ignorance. We think we are within the mark, when we calculate that one-third of those who die beyond the age of forty-five, are cut off by one or other form of these maladies, and that they constitute, as it were, the *phthisis* of middle and advanced age. The better classes of society are affected in no very unequal ratio from the lower, for although they are not so much exposed to violent bodily exertions as the latter, yet the mind is more placed upon the stretch, and the keener sensibilities conferred by education and refinement prove a source of misery and ill, as well as of salutary gratification:

"Still carried to excess in each domain,
The fav'rite good begets peculiar pain."

We know not how it is, but these diseases are too often regarded as matters of speculation and curiosity, rather than of practical importance. We think such an estimate not only a false one, but pernicious in its consequences both to patient and practitioner. It is destructive to the former, because his case is mistaken, and consequently mistreated, his sufferings aggravated, and his death too often hastened by injurious measures; and it is equally dangerous to the latter, because it either lulls him into a supine confidence, or hurries him on to fatal rashness. We are far from endeavouring or wishing to draw an overcharged picture of the evil effects of neglecting the study of diseases of the heart, nor of the ignorance respecting them that prevails amongst the mass of the profession. By far the greater proportion of the cases that daily present themselves at the hospital have been mistaken, and surely no words can convey a more cutting sarcasm than the naked fact. The sins of the medical man, both of omission and commission, are buried, to be sure, within the walls of a public establishment when his patient is transferred to it, but the matter is otherwise in private practice. Rival practitioners are disposed to do any thing but whitewash a brother's errors.

The general symptoms of morbid affections of the heart are often very distinct, but they are also frequently obscure. When the disorganization has proceeded to the extent of inducing dropsy, the indications will, in most cases, be tolerably clear to any one at all conversant with the diagnosis of disease. When, however, the dropsy is absent or inconsiderable, which it may be with serious alteration of the heart, the diagnosis is by no means so easy by the ordinary symptoms. It is this description of cases which is constantly treated for dyspepsia, hypochondriasis, asthma, or any other convenient scapegoat that may offer. It is here too that auscultation proves of essential service, and frequently detects organic disease which had passed the ken, and eluded the tact of well-informed and experienced men. We lately saw a very good instance of this in the person of a gentleman who had taken the first advice, and been assured that no organic alteration of the heart existed. A very few minutes' examination with the cylinder proved, unfortunately with too much certainty, that he laboured under passive dilatation. We have made these few remarks with the conviction that they are essentially correct, and that candour can give offence to none. We shall esteem ourselves happy if any thing we can say will stimulate more able labourers than ourselves to cultivate the vine-yard.

In our last report, we detailed two cases of what may be roughly considered as passive dilatation of the heart. We shall now give the particulars of some others of a different description.

*Case* 1. HYPERTROPHY, APPARENTLY WITHOUT MUCH DILATATION, OF THE LEFT VENTRICLE—ŒDEMA.

James West, æt. 40, a carpenter, admitted, June 16, 1829, under the care of Dr. Chambers.

Face leuco-phlegmatic and puffy—slight ascites—œdema of scrotum and legs—shortness of breath, increased on exertion, especially ascending stairs, &c.—orthopnœa—starting from sleep, which is broken and bad—pulse full, strong, and rather irregular—skin rather warm—tongue furred—bowels open—urine scanty and high-coloured.

Attacked five or six weeks ago with dyspnœa and palpitations, which have since continued—legs swelled about the same time, or shortly afterwards—enlargement of the scrotum and abdomen of a fortnight's duration. He attributes his illness to cold and wet, but has never suffered from acute rheumatism; his occupation has been an extremely laborious one.

On examination with the cylinder, the impulsion from the ventricles was considerable, the sound of both prolonged, and that of the left so rough as to approach to the *bruit de scie*. The heart's action, however, was not heard over a very extended space. The prolongation and roughening of the ventricular sound distinctly pointed out hypertrophy, chiefly of the left chamber; from the sphere of the heart's action not being greatly increased there was probably not much dilatation of the cavities, though, no doubt, that of the left ventricle was augmented to a certain extent.

*V. S. ad* ℥xij. *Potass. supertart.* ʒvj. *omn. mane. Haustus nitri, c. Tinct. digit.* ♏x. *bis die. Diæta parcissima.*

The blood drawn was not cupped or buffed, but the force of the pulse not being subdued he was bled again to ten ounces on the 19th, and, on the 22nd, was ordered eight grains of blue pill and two of root of squills for two successive nights. On the 24th, the pills were ordered to be continued every night, and the digitalis struck out from the nitre draught. The cream of tartar working him too much the quantity was reduced one-half, and on the second of July, the mouth being sore, the mercurial was discontinued. On the 8th, a belladonna plaster was applied to the precordia; and on the 15th, being free from swelling, he was made out-patient at his own desire. The conclusion of the case in the ward book, received the significant mark from Dr. Chambers of "heart hypertrophic."

The case exemplifies well the temporary relief that rest and remedial measures will confer on these patients, even when the disease has made considerable progress. Were the individual in such circumstances as to command repose both of body and mind, he might probably enjoy many years of comfort, or even attain a considerable age. In the present case, however, we dare not entertain any hopes of this kind, for the patient must return to a laborious employment, and be subjected to those domestic anxieties and discomforts that press so heavily on the minds and bodies of the lower classes.

*Case* 2. HYPERTROPHY, WITH SOME, BUT NOT EXTREME, DILATATION—ANASARCA, ASCITES—ENLARGEMENT OF THE LIVER.

W. Moon, æt. 32, servant to a brewer, admitted, June 24, under the care of Dr. Chambers.

Face sallow, rather puffy, and pallid—some ascites—œdema of legs and scrotum—shortness of breath, especially on exertion—orthopnœa—startings from sleep—action of heart violent, and communicated strongly to carotids—pulse active and strong—tongue clean—urine scanty, and depositing pink sediment—epigastric portion of the liver feels enlarged and indurated. He leads a laborious life, is exposed to alternations of temperature, and drinks more porter than is absolutely good for him.

Has suffered for many months from shortness of breath and some degree of orthopnœa, but was not decidedly ill till within the last twelve weeks, when palpitations of the heart and swelling of the legs came on. The ascites has appeared within the last fortnight; he has never had rheumatism.

℞. *Cal.* gr. iij. *Elater.* gr. $\frac{1}{4}$, *o. n. Mist. Camph.* ʒxj. *T. Hyosciam.*, *Sp. Æth. Nit.*, *Sp. Junip. c.* āā ʒss, 6*tis horis. Diæta lactea.*

In the course of two days the anasarca and ascites had almost disappeared, and the orthopnœa, as well as the dyspnœa, were relieved. On examining the chest, the action of the heart was not heard over any very great extent, but the impulsion of the left ventricle was powerful, and its sound rather dull. *Hypertrophy, without much dilatation of the ventricle.*

On the 1st of July, in consequence of the hepatic disease, he was ordered to rub in the mercurial ointment, which affected the mouth by the 10th, and was then discontinued accordingly. On that day, we find by the report that the swellings were gone, that the calomel and elaterium produced four or five motions daily, and that the action of the heart was less violent.

18th. Face puffy—pain in the epigastrium and præcordia—pulse at wrist strong—action of the heart tumultuous. On examination again with the cylinder, the sound of the left ventricle was duller and more prolonged than of the right, and its impulsion greater than before; the sound of different chambers was heard as high as the *right* clavicle, although faintly. *Hypertrophy and dilatation, particularly the former, on the increase.*

On the 20th, the calomel and elaterium were omitted, and on the 24th, the mercurial inunction was resumed. On the 27th, the dyspnœa and orthopnœa were again more severe, there was "tickling" cough, and occasionally distressing flatulence at the stomach. The camphor mixture, with the hyoscíamus, &c. were exchanged for colocynth and galbanum pills, with camphor mixture simply. A few days afterwards the anasarca returned—the face was more swollen—the functions of the stomach more disturbed—the mouth rather sore. The impulsion of the left ventricle is very great, and the sound so rough as to approach to a *bruit de soufflet.* To the right of the sternum (patient lying on his back) the sound is clearer, and the impulsion greatly diminished. *Hypertrophy and dilatation of left ventricle increasing with the march of the disease.*

℞. *Hyd. sub.* gr. iij. *ter die in form. pil. Infus. aurant. c.* ℥iss. *Potass. supert.* ʒj. *Ext. tarax.* ʒj. *Spir. æth. nit.* ʒj. *ter die, hor. elaps. post pil. sumpt.*

Aug. 1st. Worse in all respects, and has suffered from vomiting, which was arrested by an opiate draught. He was so obstinately bent upon leaving the house, evidently from the terror of dying and being examined after death, that he was dismissed on the third, in a very pitiable and hopeless condition.

Having given these cases of hypertrophy, apparently uncombined with dilatation to any amount, we shall now relate two instances of the rheumatic affection of the heart. The pericardium would seem to be affected *par excellence* in these cases, and, in most of them, the two layers of that serous membrane become adherent. The symptoms of adhesions of the pericardium have always been considered obscure, and no doubt they are so, but by bearing in mind their frequent connexion with acute rheumatism we possess a clue of no inconsiderable value in surmising their existence.

*Case* 3. ADHESIONS OF THE PERICARDIUM—DILATATION OF THE HEART—DILATATION WITH HYPERTROPHY OF LEFT VENTRICLE—ACUTE RHEUMATISM TWO YEARS PREVIOUSLY.

Henry Hall, æt. 33, a brush-maker, admitted, July 15th, under Dr. Chambers.

Face pallid and anxious—person rather emaciated—œdema of lower extremities—dyspnœa—much wheezing after exertion — orthopnœa — startings from sleep—turbulent action of the heart, which is felt and seen beating over a larger space than natural, and communicates an undulatory motion to the epigastrium—pulse 120, small and soft—skin cool—tongue slightly furred but moist—bowels open from medicine —urine free and of natural appearance.

Nearly two years ago had rheumatism for several months—has had palpitations and dyspnœa since Christmas last —subsequently entered this hospital for dropsy, which came on in January—left the house relieved, but not cured—has been getting worse during the last five weeks.

*Emplast. canth. lat. sinist. Pil. scill. c.* ℈ss. *Pil. hyd.* gr. v. *M. omni nocte sum. Inf. gent. c. Aq. pur.* āā ʒvj. *Sp. æth. nit.* ʒj. *Mag. sulph,* ʒij. *ter die. Diæta ordinar.*

17th. Seems much exhausted by frequent action of the bowels—legs much less swelled—heart palpitates much—pulse very small, weak, and frequent—skin cold—urine scanty.

*Perge, intermiss. mag. sulph. Gill of gin in pint of water daily.*

On the 20th the pulse was strong and frequent, and he complained of pain in the left side of the chest, with increased dyspnœa. He was bled to ten ozs. and an opiate prescribed on the 21st, for the blood was neither inflamed, nor the patient relieved by its abstraction. On the 22d a blister was applied to the right side of the chest, and on the 24th it was repeated, with an opiate at night, and the addition of five minims of laudanum to the original draught. On the 27th, he was evidently sinking, the surface being cold, the mind in a state of torpor, and the respiration accompanied with the râle of the dying. Brandy, &c. were administered but he died that evening.

From accidental circumstances, we had no opportunity of examining him by the stethoscope. He was considered to labour under enlargement of the heart, and Dr. Chambers suspected adhesions of the pericardium. The reasons for this suspicion, were, the rheumatic origin of the disease, and the extensive convulsion, as it were, of the intercostal spaces during the action of the organ.

*Sectio Cadaveris.* The pleuræ on either side were universally adherent; on the left, so closely that their separation was almost impracticable. The left lung was imperfectly crepitous, gorged with blood in its inferior part, with serum in its superior; it swam in water. The right lung was still more filled with blood, crepitated even more imperfectly than the left, and in several parts was so consolidated (not exactly *hepatized*,) as to sink in water. There were no tubercles in either lung. The bronchial tubes were choked with frothy fluid, and their mucous membrane was congested.

The pericardia were universally and closely united by old adhesions; the membrane was not thickened. The heart was very large—cavity of the left ventricle dilated, and its walls hypertrophied; cavities of the other chambers augmented, but their parietes little altered; valves and aorta sound.

Liver rather large and of nutmeg colour on section—kidneys somewhat knotty but healthy — head not examined.

This case is one out of many, shewing how fallacious are the symptoms usually said to indicate hydrothorax. Here were dyspnœa, orthopnœa, startings from sleep, &c. in their acmé, and yet there neither was nor could be a single drop of fluid in the pleuræ or pericardium.

*Case* 4. RHEUMATISM—HYPERTROPHY AND SOME DILATATION OF LEFT VENTRICLE — ADHESIONS OF THE PERICARDIUM?

Thomas Gilbert, æt. 15, a paper hanger, admitted May 6th, under the care of Dr. Chambers.

Person lean and aspect delicate—pain of limbs, without swelling, aggravated when hot and relieved by sweating, to which he is very subject—strong action of the heart, with flattened thorax on either side of sternum, or what is vulgarly called the "chicken breast"—pulse small and frequent—skin moist and cool—tongue a little furred—bowels open—urine free. Ill for thirteen weeks with the pains in the limbs, accompanied for the first month or five weeks with swelling; has suffered from hæmorrhoids and prolapsus for the last two years.

*Baln. tepid. ter in 7mâ. H. Sal. c. Tinct. Hyos.* ʒss. *6tis. horis.*

On the 8th, he was ordered a belladonna plaister to the præcordia, and on the 18th, he had a fresh accession of rheumatic inflammation about the first joint of the little finger of the right hand, unaccompanied with effusion into the articulation. The pulse was frequent, but soft; skin cool by day, hot at night; tongue white; bowels open. *Calomel and opium, grs.* v. *to* j. were prescribed for two nights, with *senna draught*, and on the 20th the pain and swelling were relieved, but the calomel made him sick, and a drachm of the *vin. colchici in camphor mixture* was ordered every night with the *warm bath.* On the 22nd, he complained of sore throat, and on the 25th was prescribed, *cal. gr.* iij. *op. gr.* j. for two nights. Uneasiness and pain at the epigastrium now attracted attention and were treated with relief by poppy fomentations. On the 3d of June he complained of constant and distressing pain in the back of the head and neck, with anxious, pallid countenance, and full quick pulse. He was bled to ten ozs. and the blood shewed the buffy coat. The pain in the occiput, however, persisted, and was attacked by a blister.

On the 12th, we find, by the report, that the lad was extremely nervous, and the following note is made of the examination of the heart by the stethoscope. Action of the heart heard pretty generally over the chest, (the boy is thin)—action of the right chambers little, if at all, stronger than natural—not so that of the left; impulsion of the left ventricle considerable and its sound rough and grating, indeed closely resembling the *bruit de scie*. *Hypertrophy of the left ventricle, with some dilatation of its cavity.* On the 15th, the heart's action was very violent, the *bruit de scie* distinct, and an evident pulsation was perceived in the great vessels at the root of the neck on either side. On the 19th he complained greatly of pain in the back of the head and right arm, and was ordered the tincture of hyosciamus in salines every six hours, without other medicine.

24th. Has lately had a fresh attack of rheumatic inflammation about the right ankle, from which, however, he has almost recovered—still much pain in the occipital region—distressing flatulence.

*Aq. Menth. Vir, Aq. pur.* ā̄ā. ℥v. *Mag. Carb.* ℈i. *Vin. Col.* ℨss. *bis die.*

The flatulence was mitigated by these means, but the pain in the occiput continued severe, and on the 26th a seton was inserted in the nucha, with the effect of relieving this most obstinate and unpleasant symptom. On the 6th of July the colchicum was discontinued, and in the early part of August he was made an out patient at his own request. Little alteration had occurred in the symptoms during this interval, and the only occurrence worthy of note in the treatment was the application of a blister to the left side, on account of pain there. At the time of the patient's dismissal the severer symptoms had disappeared, but his fate is probably sealed, and the disorganization of the heart will sooner or later go on to his destruction.

The existence of hypertrophy was very obvious, but the nature of the exciting cause of the disease and the extensive pulsation of the heart would incline a person to *suspect* pericardiac adhesions. We say suspect, for unfortunately no certainty attends this part of diagnosis in our present state of knowledge. Though a frequent sequela of rheumatism, adhesions of the pericardium are not to be regarded as a constant one, nor are they to be viewed as the unvarying *essence* of rheumatic affection of the heart. In a report on the diseases of the valves we shall particularly notice a case, where, although the affection was clearly referrible to rheumatism, not a particle of adhesion between the layers of the pericardium existed.

We intended to wind up the present report by some cases of marked hypertrophy and dilatation, but we fear that our space will scarcely permit us to proceed as we could wish. As the foregoing cases have been mostly instances of hypertrophy of the left ventricle unaccompanied with any very great dilatation, we shall, however, give an instance where the latter predominated.

*Case* 5. GREAT DILATATION OF THE HEART — DILATATION WITH HYPERTROPHY OF LEFT VENTRICLE—DISORGANIZATION OF THE LIVER.

Richard Collard, æt. 36, a coachmaker, admitted Aug. 19th, under the care of Dr. Chambers.

Face puffy, phlegmatic, and bilious —anasarca of lower extremities—some ascites—dyspnœa—incapability of lying on left side—strong and extended action of the heart—pulsation and undulation at the root of the neck—pulse 120, small, and rather hard—skin cool and clammy — tongue clean, bowels open—stools clay-coloured—urine dark and very scanty.

Ailing two years with loss of appetite and occasional palpitations at the heart —six months ago legs swelled, became excoriated, and discharge even at the present time—abdomen swelled about the same period—skin has been tinged yellowish for the last five or six weeks —urine has been scanty for a month. He attributes his complaints to distress of mind.

*Ung. Hyd. Fort.* ℨi. *reg. hepat. o. n. infricand. Haust. Nitri. c. Spir. Æth. Nit.* ℨi. *ter die. Potass. Supert. Pulv.* ℥ss. *o. m. Di. ordinar.*

On the 21st a scruple of jalap was added to the supertartrate every morning, and spirit lotion was ordered for the legs.

24th. Has had three paroxysms of a mixed epileptico-apoplectic character. They occurred towards night, the longest was of two hours duration, and they were followed by deep sleep. At present he looks better, and the swellings are diminished—bowels open thrice in 24 hours. On the 26th he was not so well, had cough and troubled sleep, and the bowels were not open.

*H. Senn. c. Tinct. Jal.* ℥iss. *Potass. supert. pulv.* ʒi. *o. m. loco pulv. Potass. supert. &c. Emp. Canth. lat. sinist. Rep. alia.*

On the 31st the blister was repeated and three minims of laudanum added to the diuretic draught. On the 4th Sept. the face was yellow; orthopnœa; sharp and rather full pulse. On examining the heart by the cylinder, the sound of the ventricles was rather rough, prolonged, and approximating to slight *bruit de soufflet*—impulsion considerable, especially of the left ventricle—dulness of sound on percussion over a large cardiac region. *Dilatation of the heart, hypertrophy and dilatation of left ventricle—very little, if any induration of semi-lunar valves.* There being some bruit over the clavicles, apparently in the aorta, *slight dilatation and commencing depositions were suspected.*

On the 3d the bowels being much purged and the stools bloody, he was ordered to omit the former medicines, and to take a draught of chalk mixture with five minims of laudanum after every loose evacuation. On the 4th the diarrhœa, or rather dysentery, was checked, the patient having had only one motion, not bloody, that morning. The lips were completely excoriated and excessively sore, the anasarca and ascites were less. *The nitre draught to be resumed, with the mercurial inunction—chalk mixture if the diarrhœa returned.* He now became rapidly worse, and was evidently sinking on the 6th. Brandy was administered, but he died on the 7th.

*Sectio Cadaveris.* Legs œdematous —face puffed and bilious—body much loaded with fat.

*Thorax.* About a pint, or more, of clear yellow fluid in the right side; two-thirds of that quantity in the left. Right lung gorged with serum, but not much blood; some parts more or less solid, without hepatization. Left lung œdematous, and in much the same state as the right, but not to so great a degree; no tubercles in either lung. Mucous membrane of bronchi, large and small, deeply congested, and the tubes themselves choaked with frothy serum.

About a couple of ozs. of clear yellow serum in the pericardium. Heart externally a very large one, and loaded with fat, particularly on the right side. Great dilatation of the cavities of the right auricle and ventricle, their parietes being little increased in thickness —still more dilatation of the left chambers and hypertrophy of the walls of the left ventricle, though not to an extreme degree. Valves and aorta sound.

*Abdomen.* Very little fluid in this cavity. Liver large, hard, granulated, and of the colour and appearance of yellow wax—kidneys large, pale and somewhat soapy in appearance—no ulceration of the intestines.

*Cranium.* Much transparent fluid between the arachnoid and pia mater —universally blanched, soft, and macerated condition of the brain, but no organic alteration—some clear, limpid serum in the ventricles.

The hypertrophy of the left ventricle, though shewn to be considerable on comparing the heart with a healthy one then upon the table, was scarcely so great as the powerful impulsion during life induced us to suspect. Did the fluid in the pericardium, which probably amounted at one time to three or four ounces, caricature and exaggerate the heart's action? It will be seen that the stethoscope seemed to point out a slight dilatation of the aorta, from the *bruit* and somewhat powerful stroke above the sternum. We should mention that the stroke was not *simple,* that is, single like the pulse, but double and answering to the corresponding double systole of the heart. This is sufficient to account for the error, for although it was noticed at the time yet it was conceived that the second pulsation might be merely communicated through the neighbouring parts, and not be an essential component of the indication. Laennec in his admirable work makes mention of a case, where the error was carried to a greater extent.

In 1819, says he, I was consulted in the case of a young woman, who had exhibited for eight months the general symptoms of diseased heart. I found the action of this organ regular, and accompanied by a natural degree of impulse and sound. The right and left præcordial regions sounded well on percussion; but immediately above these, the sternum as high up as the second rib, and the whole surface of the chest corresponding with the cartilages of the second, third, fourth, and fifth ribs on the left side, yielded a completely dead sound. Over the same space, the pulsations of the heart were much louder than in the cardiac regions, and were not *simple*. Notwithstanding this last circumstance, I imagined that there existed an enormous aneurism of the ascending aorta. I did not see the patient again; and she died a few months after my examination. Upon dissection, the aorta was found perfectly sound. The tumour which had destroyed the natural resonance of the chest, was the pericardium, enormously distended by sero-purulent fluid, and which extended to the top of the chest. *Third edit. Forbes's*, p. 692.

We should mention, that in the present case, the sound was much louder near the top of the sternum than in the central region of the heart, and we question much whether the fluid in the pericardium did not play the same part, though not such a first-rate one, as in Laennec's case. But this by the way.

We have thus comprised in the present report some cases of hypertrophy nearly simple, and uncombined with much dilatation — some cases of the rheumatic affection of the heart,—and lastly, a case of dilatation and hypertrophy. At a future opportunity we shall relate some interesting cases of valvular disease, and we hope to be able to make some observations and collect some facts, that may facilitate the diagnosis of the diseases of the heart.

